# Pre-treatment blood parameters as an economical predictive marker for predicting treatment response in locally advanced cervical cancer

**DOI:** 10.12688/f1000research.160308.2

**Published:** 2025-04-15

**Authors:** Abhishek Krishna, Vishnumaya N, Fathima Shada, Pooja MS, Dilson Lobo, Athiyamaan MS, Challapalli Srinivas, Sourjya Banerjee, Johan Sunny, Paul Simon

**Affiliations:** 1 Department of Radiation Oncology, Kasturba Medical College Mangalore, Manipal Academy of Higher Education, Karnataka, Manipal, India

**Keywords:** Keywords: Cervical cancer, chemoradiation, prognostic markers, pre-treatment blood parameters, treatment response.

## Abstract

**Background:**

Cervical cancer poses a significant public health challenge, particularly in low and middle-income countries. Despite advancements in treatment, the disease remains a leading cause of cancer-related deaths among women globally. Chemoradiation utilizing cisplatin has been the cornerstone therapy for locally advanced cervical cancer. Prognostic biomarkers, including hematological parameters, have emerged as valuable tools in guiding treatment decisions and predicting outcomes.

**Methodology:**

Data from patients treated between January 2021 and June 2022 were analyzed. Demographic information, histopathology, pre-treatment blood parameters, treatment details, and response assessments were collected. The parameters assessed included hemoglobin levels, neutrophil-to-lymphocyte ratio (NLR), platelet-to-lymphocyte ratio (PLR), prognostic nutritional index (PNI), and eosinophil albumin ratio (EAR). Receiver operating characteristic (ROC) curve analysis was conducted to determine optimal cut-off values for these biomarkers.

**Results:**

Of the 140 patients included, the majority had squamous cell carcinoma (92%) and were at stage II or III. Complete response to treatment was observed in 86.4% of patients. Non-responders demonstrated significantly higher levels of hemoglobin, NLR, and EAR, along with lower PNI levels compared to responders. ROC analysis revealed cut-off values for hemoglobin (< 9.5), NLR (< 2.98), PLR (> 289.26), PNI (< 37.67), and EAR (< 49.63) associated with treatment response.

**Conclusion:**

The study highlights the potential utility of pre-treatment blood parameters as predictive markers for treatment response in locally advanced cervical cancer. Lower hemoglobin, higher NLR, and EAR, along with reduced PNI, were associated with poorer treatment outcomes. Integration of these biomarkers into clinical practice could aid in treatment planning and improve patient outcomes. Further validation and prospective studies are warranted to establish the role of these biomarkers in guiding personalized treatment strategies for cervical cancer patients.

## Introduction

Cervical cancer continues to be a substantial public health problem, especially in low and middle income countries with inadequate healthcare resources. According to the GLOBOCAN 2022 report, there were 662,301 new cases of cervical cancer worldwide in 2022, with approximately 90% of deaths occurring in countries belonging to low and lower middle income category. ^1^ Cervical carcinoma is the 9 ^th^ most commonly diagnosed cancer in globally in women and the second most commonly diagnosed malignancy in women in India. ^1, 2^ In India, the majority of patients (80.9%) present at an advanced stage. ^3^ Concurrent chemoradiation (CCRT) utilizing cisplatin has been the gold standard therapeutic regimen for locally advanced cervical cancer over the last two decades. ^4^

Prognostic markers are pivotal in cancer management, guiding treatment decisions and aiding in the prediction of disease outcomes, thus profoundly impacting patient care and therapeutic strategies. In recent studies, various prognostic and predictive biomarkers have been identified in cervical cancer, and these biomarkers have improved the understanding of the disease and refining the treatment strategies. ^5^ Systemic inflammation has shown to play a critical role in cancer development and progression. IN nervical malignancy, increased systemic inflammation correlates with adverse clinical prognoses, attributed to cellular proliferation, neovascularization, resistance to therapy, and metastatic dissemination. ^6^

Simple hematological indicators such as neutrophil counts, lymphocyte counts, and platelet counts, as well as the neutrophil-to-lymphocyte ratio (NLR), platelet-to-lymphocyte ratio (PLR), serum albumin, and their combined values, can be used to indirectly identify systemic inflammation. Several studies have reported that low hemoglobin, high NLR and PLR and low Prognostic Nutritional Index correspond to an unfavorable prognosis, increased distant metastasis, and poor response to chemotherapy and radiation therapy. ^7– 11^

Hematological parameters can be easily analysed from the complete blood count reports, which invariably are done in all patients undergoing diagnostic evaluation of cervical cancer. Hence, pretreatment blood parameters represent an economical tool for predicting response to treatment in locally advanced cervical carcinoma patients. This study was conducted to assess the feasibility of utilizing pre-treatment blood parameters as markers to predict treatment response in patients with locally advanced cervical cancer.

## Methods

The participants included patients aged 18 or older with confirmed histopathological cervical cancer, who had completed the prescribed chemoradiation and brachytherapy treatment, and had a minimum follow-up of 3 months. The study followed an ambispective study design. For the retrospective component, data was obtained from hospital medical records. However, for the prospective component, informed consent was obtained from participants before data collection. Excluded were patients with metastatic or recurrent cervical cancer, incomplete treatment, lack of relevant data, hematological/cardiovascular diseases, prior malignancies, advanced kidney Issues, allergic asthma, and active infections.

The study was started after approval from the Institution Ethics Committee, Kasturba Medical College, Mangalore (IEC KMC MLR 05/2023/228 dated 18th May 2023). Data was collected by retrospectively of cases of locally advanced cervical cancer treated with chemoradiation at the hospital between January 2021 and June 2022, adhering to inclusion and exclusion criteria. Patient & Demographic information including age, stage of the disease, histopathology, pre-treatment Hemoglobin, Total Leucocyte counts, Differential Leucocyte Counts, Platelet Counts, Serum Albumin levels, treatment parameters, and response assessments were recorded from the hospital medical records. Response to treatment was assessed based on RECIST 1.1 criteria using MRI scans at 3 months after completion of treatment. ^12^ The response was recorded either as Complete Response, Partial Response, Stable Disease or Progressive Disease. Additionally, everyone who had a complete response were classified as a complete responder (CR), and everyone else who had a partial response, a stable disease, or a progressive disease was classified as a non-responder (NR).

The parameters analyzed included Hemoglobin levels, Neutrophil Lymphocyte Ratio (NLR), Platelet Lymphocyte Ratio (PLR), Prognostic Nutritional Index (PNI), and Eosinophil Albumin Ratio (EAR) and were calculated as below ^11, 13– 15^

Neutrophil−to−Lymphocyte Ratio(NLR)=Absolute Neutrophil Counts/Absolute Lymphocyte Counts

Platelet−to−Lymphocyte Ratio(PLR)=Platelet Count/Absolute Lymphocyte Count

$$\text{Prognostic Nutritional Index}=10^{*}\;\text{Serum Albumin}\;(\mathrm{gm}/\mathrm{dl})+0.005^{*}\;\text{Absolute lymphocyte count}$$

Eosinophil Albumin Ratio(EAR)=Absolute Eosinophil Count/Serum Albumin(gm/dl)

### Statistics

Demographic data were analyzed using descriptive statistics, with continuous variables represented as mean and standard deviation (SD), while categorical variables were expressed as percentages (%). The comparison between responder and non-responder groups was conducted using the student t-test to assess the means of different parameters. Receiver Operating Characteristic (ROC) curve analysis was employed to determine the area under the curve (AUC) for hemoglobin levels, Neutrophil-to-Lymphocyte Ratio (NLR), Platelet-to-Lymphocyte Ratio (PLR), Prognostic Nutritional Index (PNI), and Eosinophil Albumin Ratio (EIR) in relation to the response to chemoradiation. The ROC curve facilitated the identification of optimal cut-off thresholds for these predictive biomarkers and evaluated their sensitivity and specificity values. Group comparisons were carried out using the Chi-square test, with statistical significance set at a p value of <0.05. All analyses were performed using Jamovi version 2.3. ^16^

## Results

A total of 140 patients who satisfied the study’s inclusion requirements were assessed. The median age of the study participants was 53.5 years. Adenocarcinoma was the second most common histology observed in 11 (8%) individuals, whereas squamous cell carcinoma was the most prevalent histology in 129 (92%) of the patients. The most common stage was Stage 2 seen in 64 (45.7%) of the patients followed by Stage III in 51 (36.4%) of patients. Out of the 140 patients, 121 (86.4%) were seen to be complete responders and 19 (13.6%) were classified as non-responders. The comparison of patient characteristics between the complete responder (CR) and non-responder (NR) cohorts is depicted in Table 1. The comparison of the pre-treatment blood parameters between the complete responders (CR) and non-responder (NR) cohorts is given in Table 2.

**Table 1.** Patient characteristics between complete responders and non-responders.

Patient characteristics	Complete responders	Non-responders	P
	N=121	N=19	
**Age (Median)**	54 years	51 years	0.567
**Stage**			
I	5 (4.1%)	1(5.3%)	0.445
II	58 (47.9%)	8 (42.1%)	0.334
III	46 (38.1%)	8 (42.1%)	0.225
IVA	12 (9.9%)	2 (10.5%)	0.450
**Histology**			
Squamous Cell Carcinoma	119(96.6%)	14 (84.3%)	0.120
Adenocarcinoma	5 (3.4%)	3 (15.7%)	0.09
**EBRT**			
50Gy in 25#	86 (71.1%)	14 (73.6%)	0.786
46 Gy in 23 #	35 (28.9%)	5 (26.4%)	0.775
**Brachytherapy**			
ICRT 7.5 Gy x 3 #	96 (79.3%)	16 (84.2%)	0.453
ICRT 7 Gy x 4 #	25 (20.7%)	3 (15.8%)	0.334
**Chemotherapy**			
Cisplatin	108 (89.2%)	17 (89.4%)	0.998
Carboplatin	11 (9.1%)	1 (5.3%)	0.567
No	2 (1.75	1 (5.3%)	0.445

**Table 2.** Pretreatment hematological parameters in both the groups (NLR-Neutrophil to Lymphocyte Ratio; PLR-Platelet to Lymphocyte Ratio; PNI-Prognostic Nutritional Index; EAR-Eosinophil to Albumin Ratio, SD-Standard Deviation).

Hematological parameters	Complete responders	Non responders	P
**Hemoglobin (gm/dl)**			
Range	8.1-13.2	7-11.5	
Mean ± SD	10.9 ± 1.03	9.4 ± 1.34	<0.001
**NLR**			
Range	0.86-9.60	2.43-9.89	
Mean ± SD	2.77 ± 1.79	4.60 ± 2.21	<0.001
**PLR**			
Range	64.7-1344	62.2-2435	
Mean ± SD	353 ± 305	578 ± 602	0.113
**PNI**			
Range	24.5-58.1	29.9-43.3	0.041
Mean ± SD	41.9 ± 5.12	37.0 ± 4	
**EAR**			
Range	1.03-474	18.7-786	
Mean ± SD	53.8 ± 14.3	90 ± 27.1	<0.001

The patients categorized as non-responders manifested significantly elevated pre treatment levels of Hemoglobin, NLR and EAR in contrast to responders. However, the Platelet-to-Lymphocyte Ratio (PLR) mean values, although higher in the non-responders group, did not attain statistical significance. Non-responder patients exhibited markedly diminished Prognostic Nutritional Index (PNI) levels compared to complete responders.

The mean values for Hemoglobin, NLR, PLR, PNI, and EAR were 10.9 ± 1.03, 2.77 ± 1.79, 353 ± 305, 41.9 ± 5.12, and 53.8 ± 14.3, respectively, in the complete responders group, while in the non-responders group, they were 9.4 ± 1.34, 2.43-9.89, 578 ± 602, 37.0 ± 4, and 90 ± 27.1. Further details are provided in Table 2.

**Figure 1.**
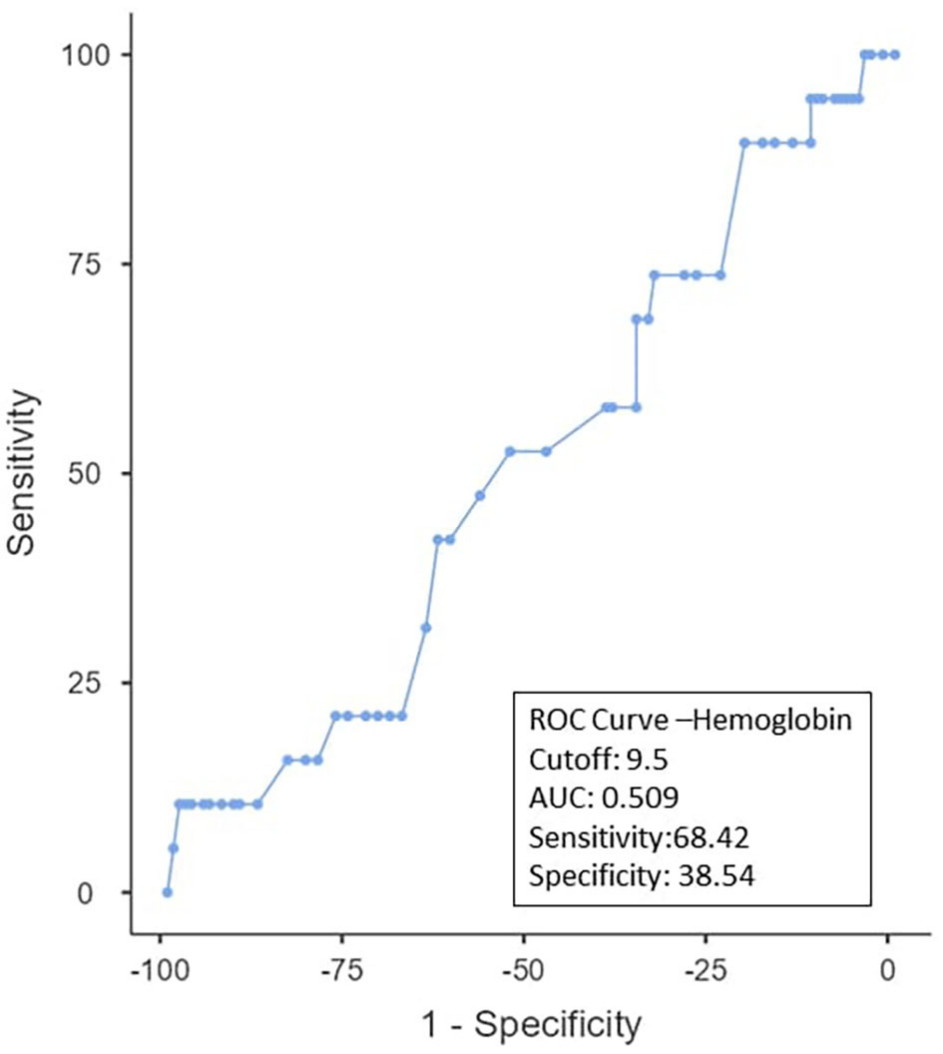
ROC curve for hemoglobin.

**Figure 2.**
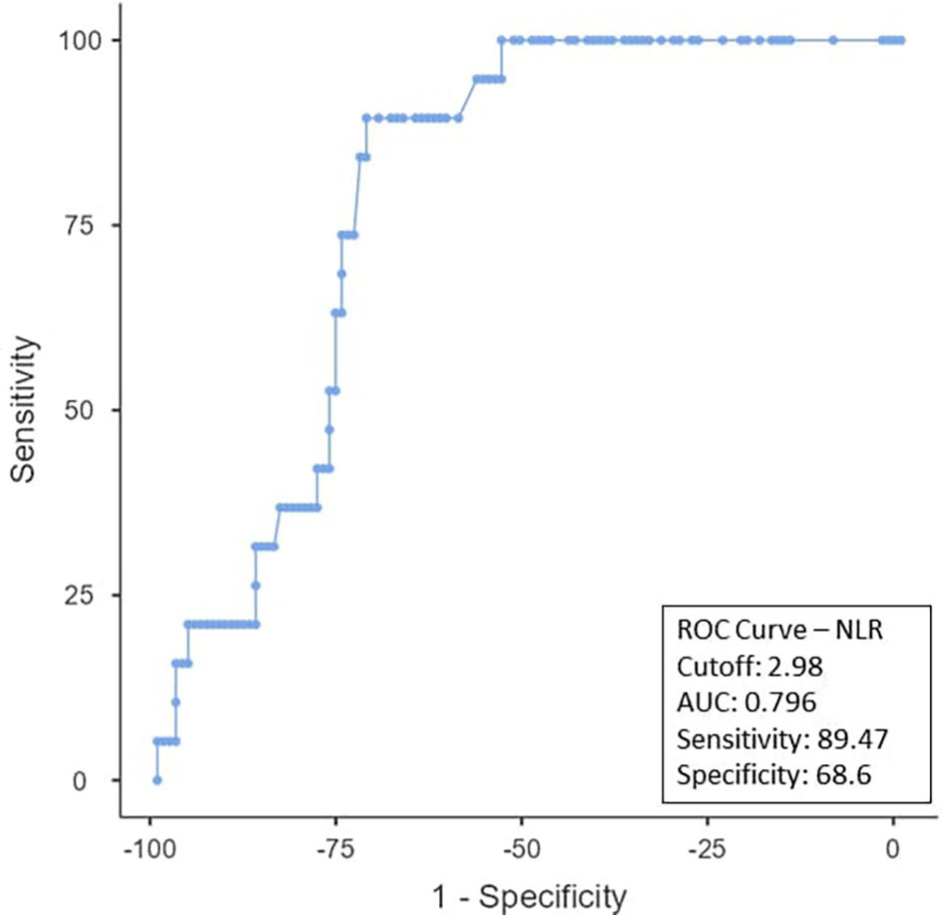
ROC curve for NLR.

**Figure 3.**
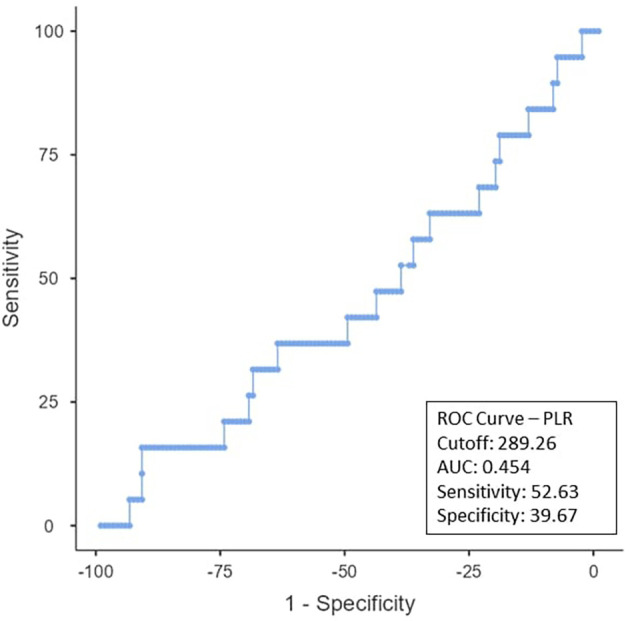
ROC curve for PLR.

**Figure 4.**
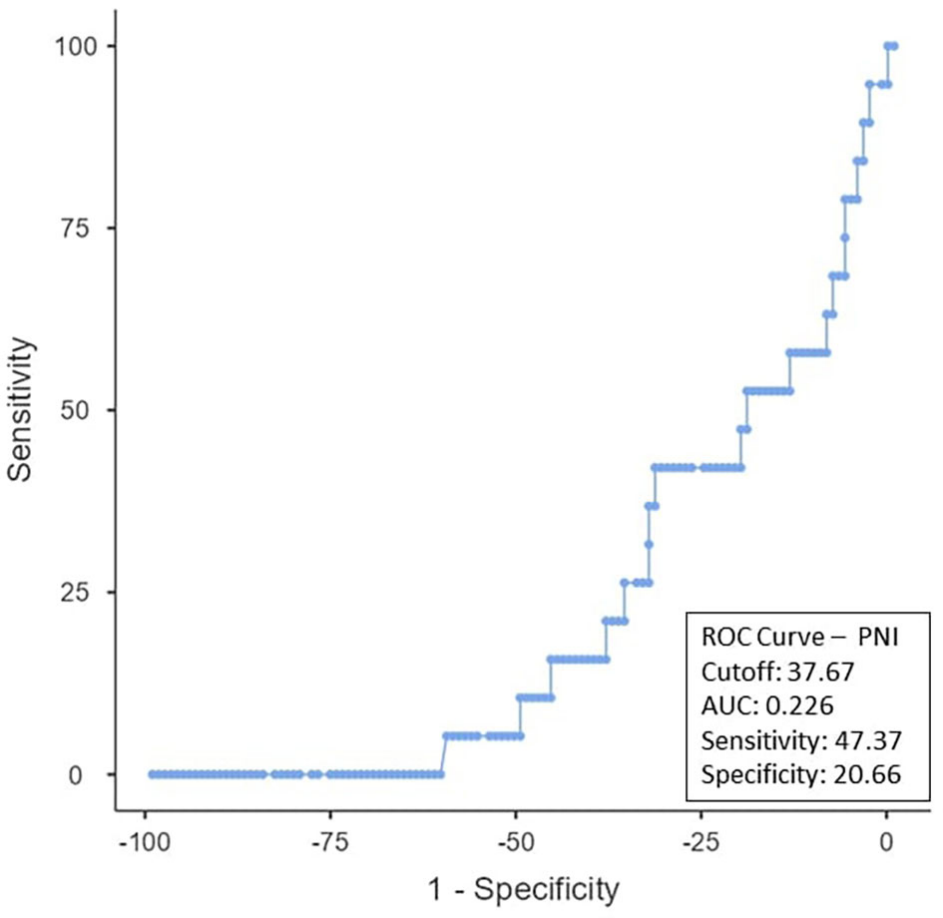
ROC curve for PNI.

**Figure 5.**
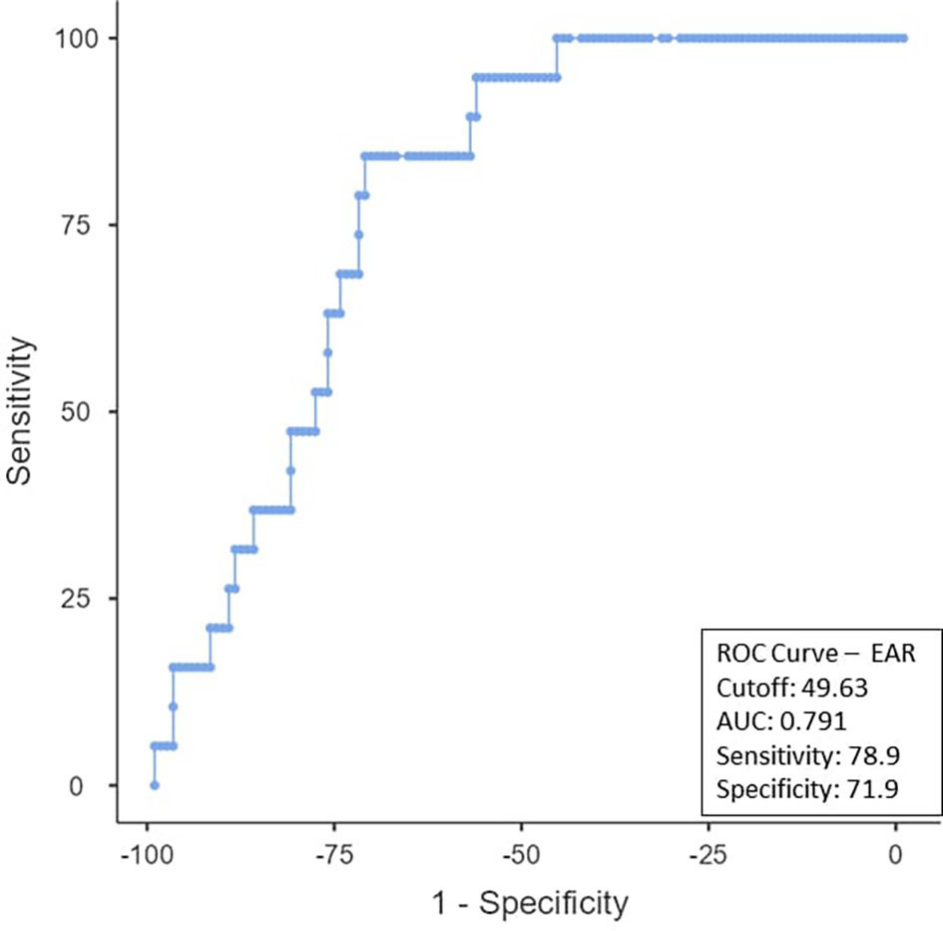
ROC curve for EAR.

To determine the ideal cut-off value for pretreatment hemoglobin, NLR, PLR, PNI, and EAR, a ROC curve analysis was performed ( Table 3). It was shown that the hemoglobin cut-off value was < 9.5 (AUC: 0.509; 95% CI), with 38.3% specificity and 68.42% sensitivity ( Figure 1). As can be observed in Figure 2, the NLR cut-off was found to be < 2.98 (AUC:0.796; 95% CI), with 89.47% sensitivity and 68.6% specificity. The PLR cut-off was 52.63% sensitive and 39.67% specific, with an AUC-0.454; 95% CI of ≤ 289.26 as shown in Figure 3. The PNI cut-off was < 37.67 (AUC-0.226; 95% CI), with a sensitivity of 47.37% and a specificity of 20.66% ( Figure 4). The EAR cut-off was < 49.63 (AUC-0.791; 95% CI) with a sensitivity of 78.9% and a specificity of 71.9% ( Figure 5). The non-responder group exhibited a notably higher proportion of patients compared to the responders group when using the calculated cut-off values from the ROC analysis ( Table 4).

**Table 3.** Cut off points for all parameters (NLR-Neutrophil to Lymphocyte Ratio; PLR-Platelet to Lymphocyte Ratio; PNI-Prognostic Nutritional Index; EAR-Eosinophil to Albumin Ratio).

Parameters	Hb	NLR	PLR	PNI	EAR
Area Under Curve	0.509	0.796	0.454	0.226	0.791
Sensitivity	68.42	89.47	52.63	47.37	78.9
Specificity	38.54	68.6	39.67	20.66	71.9
Cut off Value	9.5	2.98	289.26	37.67	49.63

**Table 4.** Comparative analysis of CR and NR using the cut-off for all parameters (NLR-Neutrophil to Lymphocyte Ratio; PLR-Platelet to Lymphocyte Ratio; PNI-Prognostic Nutritional Index; EAR-Eosinophil to Albumin Ratio).

Parameters	Complete responders N=121	Non-responders N=19	p
**Hemoglobin**			
≤9.5	10	10	<0.001
>9.5	111	9	
**NLR**			
≤2.98	85	7	0.004
>2.98	36	12	
**PLR**			
≤289.26	48	11	0.134
>289.26	73	8	
**PNI**			
≤37.67	26	10	0.003
>37.67	95	9	
**EAR**			
≤49.63	86	4	0.001
>49.63	35	15	

## Discussion

The symbiotic relationship between inflammation and cancer is deeply rooted in the history of oncology. ^17^ From Rudolf Virchow’s observations in the 19th century linking chronic inflammation to cancer development to contemporary research unveiling the molecular mechanisms underlying this association, the connection has been a focal point of scientific inquiry. ^18^ Early studies highlighted the role of inflammatory mediators in promoting carcinogenesis, while subsequent investigations elucidated the complex interplay between inflammatory cells, cytokines, and tumor cells in the tumor microenvironment. ^19^ Despite fluctuations in scientific interest over time, recent advances in cancer immunology and molecular biology have reignited enthusiasm for exploring the inflammatory pathways driving tumor progression.

Inflammatory mediators, including interleukins, tumor necrosis factor-alpha (TNF-α), and prostaglandins, modulate the behavior of cancer cells and surrounding stromal cells. Additionally, proteins, such as C Reactive protein (CRP), serum amyloid A (SAA) and other markers serve as biomarkers of systemic inflammation and are associated with poor prognosis in cancer patients. ^18, 19^ While these biomarkers have been thoroughly investigated across different cancer types, their routine availability for clinical testing is limited due to cost and accessibility constraints.

Instead, hematological parameters, readily accessible through routine blood tests, offer valuable insights into the inflammatory status of cancer patients. ^8^ Pretreatment hematological parameters indirectly provide us with an idea about the state of inflammation in the cancer patient and offer an economical alternative. ^8^ As pretreatment blood parameters are routinely conducted in all patients planned for treatment, it provides us with an opportunity to prognosticate the patient from the outset.

Changes in the composition of peripheral blood, including alterations in leukocyte subsets, platelet count, and erythrocyte sedimentation rate (ESR), reflect systemic inflammation and may serve as prognostic and predictive markers in cancer. Among these parameters, increased NLR and PLR have been associated with advanced disease stage, aggressive tumor behavior, and reduced survival in various malignancies, including cervical cancer. ^20^ The Prognostic Nutritional Index is another recent parameter that has been identified to be correlating with outcomes in cancer. By incorporating serum albumin levels, reflecting nutritional status, and absolute lymphocyte counts, representing immune competence, PNI provides valuable insights into the host’s ability to mount an effective anti-tumor response and tolerate aggressive therapies. ^21^ Low PNI values have been consistently associated with poor prognosis, including decreased survival and increased susceptibility to treatment-related complications, across various malignancies, including cervical cancer.

In the context of cancer treatment, adequate tissue oxygenation is essential for optimizing the efficacy of therapeutic interventions, including chemotherapy, radiation therapy, and immunotherapy. Low pre-treatment hemoglobin levels, indicative of anemia, have been consistently associated with reduced treatment tolerance, increased risk of treatment-related complications, and poorer outcomes in cancer patients. ^8^ Furthermore, anemia is often a consequence of underlying disease burden, inflammation, and bone marrow suppression, all of which may influence treatment response. The eosinophil-to-albumin ratio (EAR) is a less explored yet potentially insightful biomarker in the realm of cancer research and inflammation. Eosinophils can release various pro-inflammatory cytokines, chemokines, and cytotoxic granule proteins, influencing tumor growth, angiogenesis, and immune surveillance. On the other hand, albumin, a ubiquitous plasma protein, serves as a marker of nutritional status, inflammation, and disease severity. Therefore, the EAR could reflect the balance between eosinophil-mediated anti-tumor immunity and systemic inflammation in patients with cancer.

The findings of our study underscore the potential of hematological parameters as non-invasive biomarkers for risk stratification, treatment selection, and prognostic evaluation in cancer patients. Furthermore, changes in these parameters during the course of treatment may serve as dynamic indicators of treatment response and disease progression, enabling timely therapeutic interventions and personalized patient management strategies.

The observed differences in pre-treatment hematological parameters between responders and non-responders in this study may be attributed to several underlying biological mechanisms. Elevated levels of hemoglobin in responders could reflect better oxygenation and tissue perfusion, which are crucial for enhancing the efficacy of cancer treatment modalities such as chemotherapy and radiation therapy. Conversely, lower hemoglobin levels in non-responders may indicate underlying anemia, which is associated with reduced treatment tolerance and impaired oxygen delivery to tumor tissues, potentially compromising treatment efficacy.

The higher neutrophil to lymphocyte ratio (NLR) observed in non-responders suggests an imbalance between pro-inflammatory and anti-tumor immune responses. Neutrophils, as key effectors of the innate immune system, can promote tumor progression through various mechanisms, including the release of proinflammatory cytokines and suppression of anti-tumor immune responses. Conversely, lymphocytes play a critical role in tumor surveillance and cytotoxic activity against cancer cells. Therefore, an elevated NLR may reflect a state of heightened inflammation and immunosuppression, which could contribute to treatment resistance and disease progression in non-responders.

Similarly, the elevated eosinophil-to-lymphocyte ratio (EAR) in non-responders may indicate a dysregulated immune response characterized by increased eosinophilic inflammation. Eosinophils have been implicated in promoting tissue remodeling, angiogenesis, and tumor growth in various cancer types. Thus, a higher EAR may reflect an inflammatory microenvironment conducive to tumor progression and treatment resistance.

The lack of significant differences in platelet-to-lymphocyte ratio (PLR) between responders and non-responders could be attributed to the multifactorial nature of platelet function in cancer. While platelets are known to contribute to tumor progression through mechanisms such as angiogenesis and metastasis, their role in modulating treatment response may vary depending on tumor type and treatment regimen. Additionally, other factors such as concurrent medications or comorbidities may influence platelet levels and treatment outcomes, potentially confounding the observed associations.

The low prognostic nutritional index (PNI) levels in non-responders may reflect underlying malnutrition and systemic inflammation, which are known predictors of poor treatment response and survival in cancer patients. Malnutrition compromises immune function and treatment tolerance, thereby reducing the effectiveness of cancer therapy. Additionally, systemic inflammation can promote tumor progression and resistance to therapy through various molecular pathways.

Overall, the observed differences in pre-treatment hematological parameters between responders and non-responders likely reflect underlying differences in tumor biology, host immune response, and systemic inflammation. These findings underscore the potential utility of hematological parameters as prognostic markers for predicting treatment response and guiding individualized therapeutic strategies in patients with cervical cancer.

Despite the promising findings regarding the prognostic and predictive value of hematological parameters in cervical cancer, several challenges remain. Standardization of the cut-off values for Hb, NLR, PLR, EAR, PNI and other blood parameters across studies is essential to ensure consistency and reproducibility of results. Moreover, larger prospective studies with a long-term follow-up are needed to validate the utility of these biomarkers in clinical practice. Additionally, elucidating the underlying mechanisms linking inflammation and cancer progression will provide insights into novel therapeutic strategies targeting the inflammatory microenvironment.

## Conclusion

The research emphasizes the predictive significance of pre-treatment blood parameters in cases with locally advanced cervical cancer. These results have the potential to improve treatment plans and, in turn, patient outcomes. To make these metrics standard tools in clinical practice, more investigation and validation are necessary. The study emphasizes the need of further research in the field of cervical cancer management because of its possible influence on treatment response prediction and patient outcomes.

## Ethics committee approval

The study was started after approval from the Institution Ethics Committee, Kasturba Medical College, Mangalore (IEC KMC MLR 05/2023/228 dated 18 ^th^ May 2023).

This study was approved by the Institutional Review Board under protocol number IEC_KMC_MLR 05/2023/228. Data were collected from cases of locally advanced cervical cancer treated with chemoradiation at the hospital between January 2021 and June 2022.

Written informed consent was obtained from all participants.

## Data Availability

Figshare: Book1 edited with patient identifiers.xlsx DOI: https://doi.org/10.6084/m9.figshare.28060982.v3. ^22^ The project contains the following underlying data:
• Book1 edited.xlsx Data are available under the terms of the Creative Commons Attribution 4.0 International license (CC-BY 4.0).
