# Supplementary material for: Pre-treatment blood parameters as an economical predictive marker for predicting treatment response in locally advanced cervical cancer
Source: F1000Res. 2025 Apr 15;14:118. Originally published 2025 Jan 21. [Version 2] doi: 10.12688/f1000research.160308.2 (PMC12009475; doi:10.12688/f1000research.160308.2)
Supplement: Peer review history

# Reviewer report: Reviewer response for version 2

**DOI:** 10.5256/f1000research.180510.r378060

**Published:** 2025-11-08

**Authors:** Lanisha Sequeira

**Affiliations:** 1 Father Muller Medical College, Mangalore, India

Approved

Is the work clearly and accurately presented and does it cite the current literature?

Yes

If applicable, is the statistical analysis and its interpretation appropriate?

Partly

Are all the source data underlying the results available to ensure full reproducibility?

Yes

Is the study design appropriate and is the work technically sound?

Yes

Are the conclusions drawn adequately supported by the results?

Yes

Are sufficient details of methods and analysis provided to allow replication by others?

Yes

Reviewer Expertise:

Radiation Oncology, Gynaecologic Oncology, Cervical Cancer

I confirm that I have read this submission and believe that I have an appropriate level of expertise to confirm that it is of an acceptable scientific standard.

# Reviewer report: Reviewer response for version 2

**DOI:** 10.5256/f1000research.180510.r378061

**Published:** 2025-04-19

**Authors:** Agapiti H Chuwa

**Affiliations:** 1 University of Dar es Salaam, Mbeya College of Health and Allied Sciences, Mbeya, Tanzania

The issues raised in the first version have been adequately addressed by the authors in the revised version.

Is the work clearly and accurately presented and does it cite the current literature?

Yes

If applicable, is the statistical analysis and its interpretation appropriate?

Partly

Are all the source data underlying the results available to ensure full reproducibility?

Yes

Is the study design appropriate and is the work technically sound?

Partly

Are the conclusions drawn adequately supported by the results?

Yes

Are sufficient details of methods and analysis provided to allow replication by others?

Partly

Reviewer Expertise:

Gynaecologic oncology, Reproductive physiology, Molecular biology

I confirm that I have read this submission and believe that I have an appropriate level of expertise to confirm that it is of an acceptable scientific standard.

# Reviewer report: Reviewer response for version 1

**DOI:** 10.5256/f1000research.176186.r375270

**Published:** 2025-04-09

**Authors:** Lanisha Sequeira

**Affiliations:** 1 Father Muller Medical College, Mangalore, India

The authors have done a good work with respect pretreatment prognostic markers in cervical cancers. While most of the points are well written, the following observations would make the paper more robust.

 Query 1 - Introduction - In the first paragraph, 2020 GLOBOCAN is mentioned. A more recent GLOBOCAN 2022 or any latest studies post 2020 can be included.

 Query 2 - Methods Section - The type of study is not mentioned. Whether it was a retrospective analysis or a prospective one.

 Query 3 -Table 1 - The table title is wrongly written. Ideally, the title should be Patient Characteristics between two groups or should be redone for the whole population.

Is the work clearly and accurately presented and does it cite the current literature?

Yes

If applicable, is the statistical analysis and its interpretation appropriate?

Partly

Are all the source data underlying the results available to ensure full reproducibility?

Yes

Is the study design appropriate and is the work technically sound?

Yes

Are the conclusions drawn adequately supported by the results?

Yes

Are sufficient details of methods and analysis provided to allow replication by others?

Yes

Reviewer Expertise:

Radiation Oncology, Gynaecologic Oncology, Cervical Cancer

I confirm that I have read this submission and believe that I have an appropriate level of expertise to confirm that it is of an acceptable scientific standard.

# Response

**Published:** 2025-04-10

**Authors:** Dilson Lobo

**Affiliations:** Radiation Oncology, Kasturba Medical College Mangalore Manipal academy of Higher Education, Mangalore, Karnataka, India

Query 1 - Introduction - In the first paragraph, 2020 GLOBOCAN is mentioned. A more recent GLOBOCAN 2022 or any latest studies post 2020 can be included.

**Reply:** Thank you for the input. The corrections have been made and we will include recent data in the revised version of the manuscript.

 Query 2 - Methods Section - The type of study is not mentioned. Whether it was a retrospective analysis or a prospective one.

**Reply: **The study was an ambispective study. We have taken the data both as retrospective and prospective, In patients who had completed the treatment and was on follow up, the data was taken retrospectively. In patients in whom the treatment was the diagnosis was made during our study period, the data was collected prospectively.

 Query 3 -Table 1 - The table title is wrongly written. Ideally, the title should be Patient Characteristics between two groups or should be redone for the whole population.

**Reply: **The corrections have been made and will be incorporated in the revised version of the manuscript.

# Reviewer report: Reviewer response for version 1

**DOI:** 10.5256/f1000research.176186.r367720

**Published:** 2025-02-27

**Authors:** Agapiti H Chuwa

**Affiliations:** 1 University of Dar es Salaam, Mbeya College of Health and Allied Sciences, Mbeya, Tanzania

The authors are to be commended for their contribution to the growing body of knowledge on biomarkers for predicting treatment response and prognosis in cancer, an important aspect of clinical oncology. While their efforts are acknowledged, addressing the following observed issues would enhance the scientific rigour of the article:-

**1. Study design**: The study design is not clearly stated. The authors should specify the study design (e.g. retrospective case-control) to improve reproducibility.

 Also, the study used hospital medical records as the primary source of data, but the authors state that informed consent was obtained from each of the participants. Normally, consent would not be necessary for studies that use secondary (stored) data. This part needs to be clarified.

**2. Exclusion of recurrent cases**: The exclusion of recurrent cervical cancer cases is questionable. Including them could be valuable for detecting possible recurrence using haematological parameters.

**3. Results section:** The second sentence in the 'Results' section should be reordered to start with the most significant and important findings.

 Also, the author state that they have reported continuous variables as means and standard deviations, however, the variable "Age" is reported as 'median' in Table1. This part needs to be clarified.

**4. Table 1 issues: **The text description of Table 1 is inconsistent with the title. The text mentions the comparison of patient characteristics between complete responders (CR) and non-responders (NR), but the table title refers to "Patient characteristics (EBRT-External Beam Radiation Therapy)", which needs to be corrected for clarity.

 Also, the number of responders and non-responders fluctuates across cancer stages. A more detailed analysis is needed on whether the biomarkers assessed are affected by or related to the disease stage, which could help understand early signs of progression.

**5. Table 2 clarity**: The means in Table 2 are unclear. One would expect a single mean value for each category. If the authors intend to report both means and standard deviations, this needs to be clarified, particularly for the PLR values in both responders and non-responders that are abnormally large.

Is the work clearly and accurately presented and does it cite the current literature?

Yes

If applicable, is the statistical analysis and its interpretation appropriate?

Partly

Are all the source data underlying the results available to ensure full reproducibility?

Yes

Is the study design appropriate and is the work technically sound?

Partly

Are the conclusions drawn adequately supported by the results?

Yes

Are sufficient details of methods and analysis provided to allow replication by others?

Partly

Reviewer Expertise:

Gynaecologic oncology, Reproductive physiology, Molecular biology

I confirm that I have read this submission and believe that I have an appropriate level of expertise to confirm that it is of an acceptable scientific standard, however I have significant reservations, as outlined above.

# Response

**Published:** 2025-03-17

**Authors:** Dilson Lobo

**Affiliations:** Radiation Oncology, Kasturba Medical College Mangalore Manipal academy of Higher Education, Mangalore, Karnataka, India

**1. Study design**: The study design is not clearly stated. The authors should specify the study design (e.g. retrospective case-control) to improve reproducibility.

 Also, the study used hospital medical records as the primary source of data, but the authors state that informed consent was obtained from each of the participants. Normally, consent would not be necessary for studies that use secondary (stored) data. This part needs to be clarified.

**Response:** The study follows an **ambispective study design**. For the **retrospective** component, data was obtained from hospital medical records. However, for the **prospective** component, informed consent was obtained from participants before data collection.

**2. Exclusion of recurrent cases**: The exclusion of recurrent cervical cancer cases is questionable. Including them could be valuable for detecting possible recurrence using haematological parameters.

**Response:** Since our study includes only patients receiving definitive chemoradiation, most cases of recurrent cervical cancer would have already undergone prior radiotherapy. Given that repeat chemoradiation is generally unlikely in such cases, we excluded recurrent cases to maintain uniformity in our results. This ensures that our findings are specifically applicable to patients receiving first-line chemoradiation.

**3. Results section:** The second sentence in the 'Results' section should be reordered to start with the most significant and important findings.

 Also, the author state that they have reported continuous variables as means and standard deviations, however, the variable "Age" is reported as 'median' in Table1. This part needs to be clarified.

**Reponse: **We will reorder the second sentence in the 'Results' section to emphasize the most significant findings first. Regarding the reporting of continuous variables, while most were presented as means and standard deviations, age was reported as median because it is a more representative measure of central tendency for this variable, considering the distribution of age in our study population

**4. Table 1 issues: **The text description of Table 1 is inconsistent with the title. The text mentions the comparison of patient characteristics between complete responders (CR) and non-responders (NR), but the table title refers to "Patient characteristics (EBRT-External Beam Radiation Therapy)", which needs to be corrected for clarity.

 Also, the number of responders and non-responders fluctuates across cancer stages. A more detailed analysis is needed on whether the biomarkers assessed are affected by or related to the disease stage, which could help understand early signs of progression.

**Reponse:** We will modify the table title to "Patient characteristics between responders and non-responders" for clarity and consistency with the text description. A more detailed analysis will be included.

**5. Table 2 clarity**: The means in Table 2 are unclear. One would expect a single mean value for each category. If the authors intend to report both means and standard deviations, this needs to be clarified, particularly for the PLR values in both responders and non-responders that are abnormally large.

**Response:** Table 2 includes both mean and standard deviation values, which will be  mentioned in the table for better clarity.
